# Supplementary material for: Factors associated with non-attendance at appointments in the gastroenterology endoscopy unit: a retrospective cohort study
Source: PeerJ. 2022 Jul 25;10:e13518. doi: 10.7717/peerj.13518 (PMC9332409; doi:10.7717/peerj.13518)
Supplement: Supplemental Information 4 [file peerj-10-13518-s004.docx]

| Codebook to convert numbers to their respective factors | | |
| --- | --- | --- |
| Variable | Measurement level | Code |
| sex | nominal | 1 =female  0 =male |
| marital status | nominal | 1 =married  0 =single |
| intervention | nominal | 1 =ERCP  2 =Gastroscopy  3 =Colonoscopy  4= Liver Biopsy |
| appointment outcome | nominal | 1 =adherent  0 =non-adherent |
| referring source | nominal | 1=gastroenterology  0 =other |
| Anesthesia | nominal | 1= anesthesia  0 =sedation |
| GEO DİSTANCE | scale |  |
| Travel time (s) | scale(second) |  |
| Travel time (min) | Scale (minute) |  |
| Lead time | Scale (month) |  |
| Season of ref. | nominal | 1 =Spring  2 =Summer  3 =Fall  4= Winter |
| malignancy | nominal | 1 =present  0 =absent |
